# Sex Differences in the Complexity of Healthy Older Adults’ Magnetoencephalograms

**DOI:** 10.3390/e21080798

**Published:** 2019-08-15

**Authors:** Elizabeth Shumbayawonda, Daniel Abásolo, David López-Sanz, Ricardo Bruña, Fernando Maestu, Alberto Fernández

**Affiliations:** 1 Centre for Biomedical Engineering, Department of Mechanical Engineering Sciences, Faculty of Engineering and Physical Sciences, University of Surrey, Guildford, GU2 7XH, UK; 2 Laboratorio de Neurociencia Cognitiva y Computacional, Universidad Politécnica de Madrid—Universidad Complutense de Madrid (UPM-UCM), 28223 Madrid, Spain; 3 Departamento de Psicología Experimental, Procesos Cognitivos y Logopedia, Universidad Complutense de Madrid, 28223 Madrid, Spain; 4 Networking Research Center on Bioengineering, Biomaterials and Nanomedicine (CIBER-BBN), 28029 Madrid, Spain; 5 Departamento de Medicina Legal, Psiquiatría y Patología, Universidad Complutense de Madrid, 28040 Madrid, Spain

**Keywords:** permutation Lempel-Ziv complexity, magnetoencephalography, source space, healthy aging, sex differences

## Abstract

The analysis of resting-state brain activity recording in magnetoencephalograms (MEGs) with new algorithms of symbolic dynamics analysis could help obtain a deeper insight into the functioning of the brain and identify potential differences between males and females. Permutation Lempel-Ziv complexity (PLZC), a recently introduced non-linear signal processing algorithm based on symbolic dynamics, was used to evaluate the complexity of MEG signals in source space. PLZC was estimated in a broad band of frequencies (2–45 Hz), as well as in narrow bands (i.e., theta (4–8 Hz), alpha (8–12 Hz), low beta (12–20 Hz), high beta (20–30 Hz), and gamma (30–45 Hz)) in a sample of 98 healthy elderly subjects (49 males, 49 female) aged 65–80 (average age of 72.71 ± 4.22 for males and 72.67 ± 4.21 for females). PLZC was significantly higher for females than males in the high beta band at posterior brain regions including the precuneus, and the parietal and occipital cortices. Further statistical analyses showed that higher complexity values over highly overlapping regions than the ones mentioned above were associated with larger hippocampal volumes only in females. These results suggest that sex differences in healthy aging can be identified from the analysis of magnetoencephalograms with novel signal processing methods.

## 1. Introduction

Although sex differences in brain structure and functioning are a matter of crucial importance, they remain relatively unexplored [1]. Two main reasons were posed to highlight the importance of this issue: the differential prevalence of several neuropsychiatric disorders and the presence of some distinctive cognitive and behavioral traits. For example, Alzheimer’s disease, major depression, and anorexia nervosa are more prevalent in females, while autism spectrum disorders, dyslexia, schizophrenia, or attention deficit/hyperactivity disorder are more prevalent in males [2,3,4,5,6,7]. On the other hand, performance on some tasks such as mental rotation or the embedded figures test is generally higher in males, while scores of verbal memory or verbal fluency are usually higher in females [8]. Furthermore, personality/behavioral traits such as physical aggression appear more frequently in males, whereas neuroticism and agreeableness are more common in females [9,10,11].

A recent meta-analysis [12] of macrostructural sex differences revealed that the above-presented tendencies may have an underlying neurobiological basis. The authors reported significant differences in volume and tissue density that affected regions such as the amygdala, hippocampus, putamen, insula, cingulate gyrus, frontal pole, posterior cingulate/precuneus, planum temporale, etc. Importantly, Ruigrok and co-workers [12] pointed out that the observed differences involved regions that are part of the limbic and language systems. Males tended to show larger volumes in limbic regions and posterior cingulate/precuneus, whereas females exhibited larger volumes in language regions and anterior cingulate. In addition to structural measures, functional investigations by means of MRI (i.e., fMRI), such as those assessing brain connectivity, also showed significant differences. For example, the strength of the connectivity within the default mode network (DMN) was higher in females, while the connectivity within the sensorimotor and visual cortices was stronger in males [13].

Neurophysiological techniques such as electroencephalography (EEG) or magnetoencephalography (MEG) are consistent alternatives to fMRI due to their superior temporal resolution and direct measurement of neural activity. EEG studies that assessed power values in traditional frequency bands reported contradictory results. For instance, those that focused on the alpha band revealed either no differences between sexes [14], higher power in males [15], or higher power in females [16]. Similar discordant findings were reported for the theta and beta bands. With respect to coherence or correlation analyses, some investigations found higher values in males as compared with females, especially among children [14,17].

In spite of the usefulness of linear methods based on the Fourier transform for the characterization of sex-related changes in brain activity recorded in EEG and MEG signals, the highly complex nature of these recordings is probably better analyzed using methods that address the non-linear dynamics. In this regard, the first studies investigating sex differences in complexity or entropy estimates reported higher values in females [18,19]. More recent investigations [20,21] supported this notion by calculating approximate entropy (ApEn) and fractal dimension (FD) values. Our group performed an exhaustive investigation of complexity evolution across the life span, using Lempel-Ziv complexity (LZC) as an estimator of complexity and MEG as a scanning technique [22]. LZC presents some advantages as compared with other classical methods such as correlation dimension (D2) or first Lyapunov exponent (L1), as it can be calculated even for short data segments and in non-stationary signals; consequently, it is better suited for the analysis of the electromagnetic brain activity [23]. Results demonstrated a clear sex effect with females exhibiting higher LZC scores in central and posterior regions [22].

Recently, new algorithms of symbolic dynamics analysis were introduced to estimate complexity in biomedical signals. For example, permutation Lempel-Ziv complexity (PLZC) is a method that combines concepts from permutation entropy [24] with the classic LZC complexity algorithm and presents some advantages when compared with the original LZC algorithm [25], namely, the ability to reflect the changes in the relationships between signal samples in ways that are not possible with LZC. Therefore, the analysis of MEG signals with PLZC could provide additional insights into sex differences with healthy aging. Moreover, all the above-cited EEG or MEG studies were performed in the electrode/sensor space, and no anatomical information was available on the sources of the observed brain activity. Current source localization models for MEG signals reached a notable evolution and allow a sufficiently precise definition of the anatomical origin of the signals of interest. 

With all these considerations in mind, we carried out an investigation aimed at assessing sex differences with PLZC within a sample of elder healthy subjects. It was hypothesized that PLZC would identify differences in complexity due to sex in MEGs recorded during resting state. The study of the resting state is relevant as it is possible that subtle differences in the background MEG activity of males and females (which could be otherwise hidden during tasks) exist and, therefore, might give further understanding of the sex differences associated with healthy aging (see below). A source reconstruction algorithm was applied to MEG signals in order to obtain an image of complexity distribution along the brain. PLZC scores were correlated with volumetric data derived from the hippocampal region due to its key role in aging and dementia. To the best of our knowledge, this is the first study that offers such a combination of functional and neuroanatomical information. 

## 2. Materials and Methods 

### 2.1. Subjects

Subjects were recruited from three different centers, namely, the Neurology Department at Hospital Universitario San Carlos, the Center for Prevention of Cognitive Impairment, and the Seniors Center of Chamartin District located in Madrid, (Spain). A total of 98 subjects (49 males, 49 female) aged 65–80 (with an average age of 72.71 ± 4.22 for males and 72.67 ± 4.21 for females) took part in this study. All subjects were age-matched, with no significant differences between the sexes (*p* > 0.05). 

### 2.2. Diagnosis Criteria

Exclusions used in the compilation of the sample making up the database used in this study included the following:

(1) No history of psychiatric or neurological disorders or drug consumption that could affect MEG activity, such as cholinesterase inhibitors;
(2) No evidence of infection, infarction, or focal lesions in a T2-weighted scan within two months of MEG acquisition;
(3) A modified Hachinski score and Geriatric Depression Scale short form score ≥5;
(4) No history of alcoholism, or chronic use of anxiolytics, neuroleptics, narcotics, anticonvulsants, or sedative hypnotics;
(5) No B12 vitamin deficit, diabetes mellitus, thyroid problems, syphilis, or human immunodeficiency virus (HIV).

### 2.3. MEG Signal Recording and Pre-Processing

Resting-state MEG signals were recorded at the Laboratory of Cognitive and Computational Neuroscience (Madrid, Spain). Four minutes of MEG data were acquired using a 306-channel (102 magnetometers, 204 planar gradiometers) Vectorview MEG system (Elekta AB, Stockholm, Sweden), while subjects sat comfortably with eyes closed inside a magnetically shielded room (VacuumSchmelze GmbH, Hanau, Germany). During signal acquisition, four head position indication (HPI) coils were placed on each subject’s scalp (two on the mastoids and two on the forehead) to enable continuous head position estimation, which was used to track head movements. Moreover, a vertical electrooculogram of the left eye was used to capture blinks and eye movements. MEG data were acquired using a sampling rate of 1000 Hz and an online anti-alias bandpass filter (0.1 and 330 Hz). Recordings were processed offline to remove magnetic noise originated outside the head with a spatiotemporal signal space separation algorithm [26], using as parameters a correlation window of 0.9 and a time window of 10 s. Ocular, muscular, and jump artefacts were firstly identified using an automatic procedure from the Fieldtrip package [27], and then visually confirmed by a MEG expert. In addition, an ICA-based procedure was employed to remove the electrocardiographic component when it was clearly identified, after which the remaining data were segmented into four-second epochs of artefact-free activity (average number of epochs used: 47.5 ± 8.7). Due to data redundancy after the spatiotemporal filtering, only magnetometer data were used in the subsequent analysis [28]. Clean trials were padded with two seconds of real signal on both sides prior to the application of a bandpass filter using a finite impulse response filter (2000th order, built using Hamming window) between 2 and 45 Hz to prevent edge artefacts.

### 2.4. MRI Acquisition

T1-weighted MRIs (acquired in a General Electric 1.5-T magnetic resonance scanner, using a high-resolution antenna and a homogenization PURE filter (Fast Spoiled Gradient Echo sequence, TR/TE/TI = 11.2/4.2/450 ms; flip angle 12°; 1 mm slice thickness, 256 × 256 matrix, and FOV 25 cm) were available for each subject. In order to acquire the volumes of several brain regions, the specialized automated tool for cortical and subcortical segmentation [29] available in the FreeSurfer software (version 5.1.0) was used during pre-processing stages. Moreover, due to their association with characterizing MCI and AD progression, hippocampal volumes were selected for use as anatomical evidence of brain atrophy [30]. In addition to this, brain region volumes were normalized with respect to the overall intracranial volume (ICV) so as to account for differences in head volume over subjects.

### 2.5. Source Reconstruction

The source model used was a modified version of the Harvard–Oxford atlas with 1485 cortical sources placed in a homogeneous grid of 1 cm in the MNI template, after which each source was linearly transformed into subject space using the individual T1-weighted scan. Following the linear transformation, each source belonging to one of the 64 areas of the reduced Harvard–Oxford atlas was labeled [31]. The lead field was then computed using a realistic single shell model [32]. Lastly, to obtain the source time series, a linearly constrained minimum variance (LCMV) beamformer [33] was employed to compute a spatial filter from the lead field and the epoch-averaged covariance matrix. The selection of a beamformer over other techniques such as minimum norm estimates is based on its larger versatility when dealing with source specific parameters as power estimations [34].

### 2.6. Complexity Calculation

As briefly advanced in the introductory section, PLZC is a modification of the LZC algorithm that uses permutations (motifs) of a chosen length *m* to estimate the complexity of a signal instead of applying a coarse-graining procedure to the signal [25]. According to Bai and colleagues, different combinations of motif length (*m*) and time delay (*τ*) can be used to estimate the complexity of a signal, with their selection being dependent on the nature and length of the signal being investigated. Smaller values of *m* are better suited (when used in combination with low values of *τ* such as *τ* = 1) to investigate short signals with fast-changing dynamics, while larger values of *m* are suitable for longer signals with slower-changing dynamics. Thus, the selection of the ideal motif length and time delay is very dependent on the signal acquisition parameters, as well as the signal dynamics. 

To estimate complexity using the PLZC algorithm, the number of possible motifs given by m! in the permutation symbolizing procedure must be less than the time series length α. The total number of sub-sequences in the original time series is given by Equation (1) [25,35].

(1) $$L\;\left(n\right)=c\left(n\right)\left[log_{m!}\left\{c\left(n\right)\right\}+1\right]$$

where *c*(*n*) is the complexity counter of symbol sequence estimated using the LZC algorithm [36]. PLZC can be defined as a normalized *L*(*n*) as follows, where *n* represents the total length of the time series and m!≤α:

(2) $$PLZC=\frac{c\left(n\right)\left[log_{m!}\left\{c\left(n\right)\right\}+1\right]}{n}$$

However, in the event of signals with many samples (usually >9!), PLZC can be simplified and defined as follows:

(3) PLZC=c(n)[logm!n]n

As the effects of increasing *τ* are like the effects of down-sampling the data, a more conservative approach of using *τ* = 1 ensures that all data points in the time series data are used. Moreover, the use of conservative values of τ in combination with high values of m ensures that the frequency content in the data is adequately investigated (without the risk of violating Nyquist criteria, especially in the event of prior down-sampling before data analysis) [37,38]. After normalization, the values of PLZC range between 0≤PLZC≤1, where the lower limit reflects regularity in the signal, and the upper limit reflects irregularity in the signal [25].

It must be noted that, to assist with the selection of an adequate value of *m*, color maps were plotted to act as a visual qualitative tool during the sensor space analysis. Embedding dimensions of four, five, and six were investigated, after which an embedding dimension of *m* = 5 was used as this managed to capture changes in the MEG signals. Moreover, as the effect of changing the values of τ is similar to that of down-sampling the data, the value of τ was set to *τ* = 1 for all calculations in this study so as to ensure the capture of all the changes in the data [39]. Lastly, as the data were already segmented into four-second epochs, no further segmentation was performed.

### 2.7. Statistical Analyses

In this study, preliminary analyses were performed in broadband, using sensor space analysis, as a first step in identifying channels over regions of the brain with differing PLZC between males and females. Following this investigation, more detailed analyses in source space were then performed using PLZC in the broadband (2–45 Hz), as well as the theta (4–8 Hz), alpha (8–12 Hz), low beta (12–20 Hz), high beta (20–30 Hz), and gamma (30–45 Hz) bands.

Analyses to identify the cortical sources of the significant differences between males and females were performed using independent *t*-tests and the Monte Carlo method, with corrections for multiple comparisons done using cluster-based permutation tests (CBPT) [40] with 10,000 repetitions. Correlation coefficients between the source complexity values and gray matter volume data (such as bilateral hippocampus, parahippocampus, and entorhinal volumes) were also calculated for each source separately and corrected again using CBPT to identify associations between the PLZC values and gray matter atrophy. In this study, probabilities with a significance of *p* < 0.05 after multiple comparisons correction were considered as significant.

The procedure for the cluster-based permutation test incorporated seven steps. Firstly, the source-level activity was compared across groups, individually for each position, using a simple statistic (in our case, between-group *t*-test and Pearson correlation test). Secondly, the participants were relabeled into a series of 10,000 random partitions, and the statistic was calculated again for each one of these permutations. Thirdly, the permutation data were used to build a nonparametric null distribution, individually for each source position. Fourthly, the original statistics were compared to these null distributions using an alpha value of 0.05, and the significant source positions were clustered according to their spatial contiguity. Fifthly, a single cluster-based statistic was calculated, by summing the value of the statistic for each of the source positions belonging to the cluster. Sixthly, the same procedure was applied to each one of the random partitions, generating a null distribution of cluster-based statistics. Finally, the original cluster-based statistic was compared to this null distribution, resulting in a *p*-value indicating the statistical significance of the cluster.

## 3. Results

**Figure 1:**
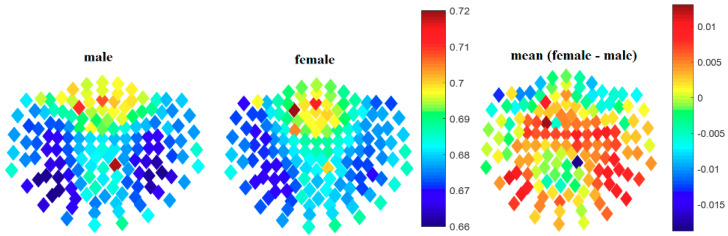
Illustration showing the broadband sensor space permutation Lempel-Ziv complexity (PLZC) values for each channel (shown as diamonds) in males and females. Although the differences were not significant (*p* > 0.05), the cluster of sensors over the central region of the brain had higher values in females than in males.

The first part of our study was to perform a sensor space analysis to enable quick identification of the channels over regions of the brain with higher PLZC than others, as well as to identify the sex with higher complexity values. Like our previous work [39], the highest complexity values were identified in the frontal (anterior) sensors for both sexes, with males having higher anterior values than females (shown in Figure 1). Nevertheless, females were found to have higher mean complexity values in sensors over the antero-central regions of the brain when compared to their male counterparts.

**Figure 2:**
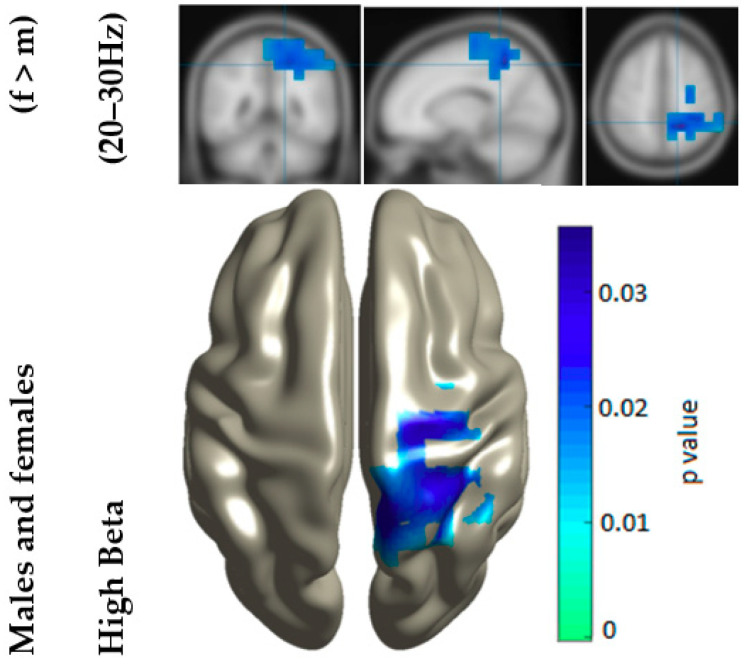
Illustration of the location of the cluster of sources, including the precuneus, with significantly different complexity values (*p* = 0.0352, Cohen’s *d* = 0.1217) between the males and females in the high beta frequency band. The significant cluster was interpolated on a surface template (as seen in the lower part of the image), while the coronal, sagittal, and axial brain views are shown on interpolated T1-weighted images (as seen in the upper part of the image).

Following the sensor space broadband analysis, a source space complexity analysis was performed to investigate these differences further, both in broadband and narrow bands, after which a source-by-source statistical comparison, using CBPT to control for multiple comparisons, was carried out to identify the exact location of the cluster containing the significant differences between the sexes. Like the sensor space analysis, broadband comparisons did not show any significant differences between males and females with *p* > 0.05. However, in the narrowband analyses, significant differences were observed in the high beta band (*p*_high_beta_ = 0.0352, Cohen’s *d* = 0.1217), but not for other frequencies (*p*_theta_ = 0.1884, *p*_alpha_ = 0.5031, *p*_low___beta_ = 0.2170, *p*_gamma_ = 0.7435) as shown in Figure 2. A significant cluster existed (in the high beta band ranging between 20 and 30 Hz) in the region that coincided with where the highest differences were observed in the sensor space analysis (Figure 1). In addition to this, females were found to have significantly higher complexity values than males (*p* = 0.0352) in regions including the precuneus, superior parietal lobe, and superior occipital cortex.

**Figure 3:**
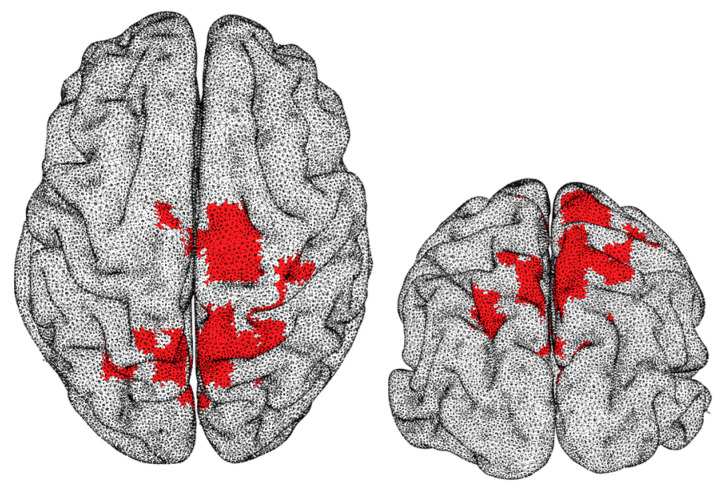
Illustration of the central cluster with complexity values, in the high beta frequency range (20–30 Hz), which significantly correlated (average rho = 0.4343, *p* = 0.022) with right-hippocampal volume scores for females.

To enable further understanding of the meaning of the PLZC results, correlations between complexity values and brain structure volume were evaluated. The results from this analysis in the whole sample did not bring any significant association between both variables in our whole sample (i.e., when considering males and females together). Thus, intra-group correlation analyses were then performed to identify if changes in brain volumes were related to changes in complexity values for the individual sex groups. Interestingly, correlation results were different in both groups. For males, no significant correlation was observed between complexity values and brain structure volumes. However, for females, a significant cluster was identified. It is remarkable that this cluster was mainly formed by regions overlapping those observed to have significant differences shown in Figure 2. Figure 3 shows an illustration of the cluster which was positively correlated (average rho = 0.4343, *p* = 0.0220) to the right-hippocampal volumetric scores, thus highlighting the association between greater high beta complexity values and brain structure volumes in the female group. 

## 4. Discussion

PLZC was used in this pilot study to evaluate the complexity of MEG signals in source space. Complexity was estimated in narrow bands (i.e., theta (4–8 Hz), alpha (8–12 Hz), low beta (12–20 Hz), high beta (20–30 Hz), and gamma (30–45 Hz)), with further statistical analyses to determine the associations between complexity values and brain structural volumes performed. Results showed that healthy females had significantly higher complexity values than males in posterior brain regions including the precuneus, and the parietal and occipital cortices. Moreover, our correlation analysis showed that higher complexity values over highly overlapping regions to the ones mentioned above were associated with larger hippocampal volumes only in females.

Different techniques were applied over the years to acquire more information related to the differences between males and females on the activity of the brain [39,41,42,43,44]. Detection of these sex differences is not a trivial task as male and female activity in MEG signals is very similar, especially for healthy subjects. Nevertheless, in this study, significant sex differences were observed in a cluster located in the central and posterior regions of the brain. Such findings are in agreement with the increase of ApEn values over parieto-occipital sensors within females in the EEG reported by Jausovec and Jausovec [20], and the FD results obtained by Ahmadi and colleagues [21]. Similarly, a previous MEG study revealed higher LZC scores in central and posterior sensor groups in females [22]. However, our findings, using a source reconstruction algorithm, allow for a better understanding of the anatomical basis by adding precise information about the spatial location of these differences. In this vein, the significant complexity differences observed in the precuneus deserve further consideration. The precuneus is associated with memory retention and linked to early Alzheimer’s disease (AD) detection [45,46,47]. Moreover, the precuneus is part of a region making up one of the main hubs of the brain functional network, which also acts as a critical hotspot of the default mode network (DMN) [48]. Interestingly, a very recent MRI investigation by Ritchie and co-workers [49] reported increased tract complexity and connectivity in the DMN within females. This is a research of particular importance, as an investigation by our group revealed a significant positive correlation between white-matter (WM) connectivity and LZC [50]. The investigation demonstrated a quadratic relationship between age and complexity, with a brisk increase from infancy to adolescence followed by a sustained augment that reached a plateau. Once the maximum was reached, complexity scores tended to show a slow decrease during senescence. These results were interpreted in terms of a close relationship between functional complexity and WM structure. Such an interpretation was recently supported by Farahibozorg and colleagues [51] in an MRI study where the FD of WM structures was calculated. FD evolution along the age range of the sample resembled that observed by Fernández and colleagues [50], and female FD scores were higher as compared with males. 

Interestingly, the current results expand previous affirmations posed by Fernández et al. [50]. In that piece of research, it was claimed that power spectral measures would be more correlated with gray-matter structure, while complexity measures would be more associated with WM structure. In this new study, an investigation to correlate complexity values with the volumes of some brain structures was performed. Correlation analyses with hippocampal volumes showed that female complexity values were positively correlated with right-hippocampus volumes, while this was not the case for males. This particular finding emphasizes the relevance of complexity changes across the lifespan, as the hippocampus is a critical region during normal and pathological ageing. This finding supports the notion of a strong relationship between functional complexity and the neuroanatomy of the brain [52,53]. Such a relationship is also of interest as the hippocampus is a brain area which is critical for learning and memory and, thus, is especially vulnerable to damage at early stages of AD. In turn, changes in complexity were linked to characteristics of the early AD continuum [54]. Results found in the current investigation were observed in a group of healthy elders and, consequently, it might be questioned whether an increased complexity accompanied by larger hippocampi represents a sign of better-preserved brain functioning, even though our findings seem to suggest so. 

Several investigations (see, for example, References [55,56,57]) showed lager hippocampal volumes in females as compared with males. In addition, Lehman and colleagues [58] demonstrated that aged females seemed to be less affected by the deleterious effects of APOE4 on brain structure and cognitive performance. This line of evidence agrees with early research on sex differences in cognitive aging that suggested female advantages in domains such as episodic memory [59], although more recent studies reported similar rates of cognitive decline [60,61]. Proust-Lima and colleagues [62] claimed that such a discordance might be due to differences in the age distribution of the samples and proposed that, at older ages, females show a steeper decline than males in several domains that coincided with a higher incidence of AD (see also Reference [54]). Therefore, although the underlying mechanism driving these changes remains elusive [63], it is possible that our current results represent a stage where the female’s brain is structurally and functionally better preserved. That stage might precede the phase of steeper decline observed in some studies. In fact, Fernández and colleagues [22] reported higher complexity within females along the age distribution of the sample, with the exception of the oldest participants. Within this particular group, males exhibited larger LZC scores than females.

Although the results of this study are promising, there are some limitations worth mentioning. While PLZC is a new method that shows promise for the analysis of the complexity of brain activity, its parametric nature (i.e., the results depend on the choices of input parameters) means that different combinations of input parameters could reveal additional information about the brain activity. This should be investigated in future studies. Furthermore, the analysis should also be extended to MEG signals from patients with AD and mild cognitive impairment in order to validate the usefulness of the results presented herein. Lastly, as any analysis using multi-channel MEG data, results might be partly affected by volume conduction. In this sense, the only expected effect when analyzing LZC is a small blurring of the results, making differences less localized and slightly more diffuse across the main area responsible of the differences. Although this effect does not compromise the reliability of the results, it should be considered when interpreting the exact location of the differences.

## 5. Conclusions

In summary, results from this study indicated that MEG signals from females have significantly higher complexity (PLZC) values than those from males in posterior brain regions including the precuneus, and the parietal and occipital cortices. Moreover, for females, there is a significant correlation between better preserved gray-matter integrity and higher complexity values. Thus, similar to conclusions reported in precedent studies (see above), sex should be considered as an independent distinguishing characteristic when studying different populations. This pilot study supports the usefulness of new algorithms to estimate complexity in biomedical signals based on symbolic dynamics to that aim.
